# Effect of early-stage autophagy inhibition in BRAF^V600E^ autophagy-dependent brain tumor cells

**DOI:** 10.1038/s41419-019-1880-y

**Published:** 2019-09-12

**Authors:** Shadi Zahedi, Brent E. Fitzwalter, Andrew Morin, Sydney Grob, Michele Desmarais, Anandani Nellan, Adam L. Green, Rajeev Vibhakar, Todd C. Hankinson, Nicholas K. Foreman, Jean M. Mulcahy Levy

**Affiliations:** 1 Department of Pediatrics, University of Colorado Denver, Aurora, CO USA; 2 The Morgan Adams Foundation Pediatric Brain Tumor Research Program, Children’s Hospital Colorado, Aurora, CO USA; 3 Department of Pharmacology, University of Colorado Denver, Aurora, CO USA; 4 Department of Neurosurgery, University of Colorado Denver, Aurora, CO USA

**Keywords:** CNS cancer, Paediatric cancer

## Abstract

Autophagy is a multistage process. Progress within the field has led to the development of agents targeting both early (initiation) and late (fusion) stages of this process. The specific stage of autophagy targeted may influence cancer treatment outcomes. We have previously shown that central nervous system (CNS) tumors with the *BRAF*^*V600E*^ mutation are autophagy dependent, and late-stage autophagy inhibition improves the response to targeted BRAF inhibitors (BRAFi) in sensitive and resistant cells. Drugs directed toward initiation of autophagy have been shown to reduce tumor cell death in some cancers, but have not been assessed in CNS tumors. We investigated early-stage inhibition for autophagy-dependent CNS tumors. BRAFi-sensitive and resistant AM38 and MAF794 cell lines were evaluated for the response to pharmacologic and genetic inhibition of ULK1 and VPS34, two crucial subunits of the autophagy initiation complexes. Changes in autophagy were monitored by western blot and flow cytometry. Survival was evaluated in short- and long-term growth assays. Tumor cells exhibited a reduced autophagic flux with pharmacologic and genetic inhibition of ULK1 or VPS34. Pharmacologic inhibition reduced cell survival in a dose-dependent manner for both targets. Genetic inhibition reduced cell survival and confirmed that it was an autophagy-specific effect. Pharmacologic and genetic inhibition were also synergistic with BRAFi, irrespective of RAFi sensitivity. Inhibition of ULK1 and VPS34 are potentially viable clinical targets in autophagy-dependent CNS tumors. Further evaluation is needed to determine if early-stage autophagy inhibition is equal to late-stage inhibition to determine the optimal clinical target for patients.

## Introduction

Macroautophagy (referred to hereafter as autophagy) plays a critical role in maintaining cellular homeostasis by eliminating damaged organelles and misfolded proteins. It functions through a multistage degradation process which can be organized into five distinct phases: initiation, elongation, closure, maturation, and degradation^1,2^. Initiation, the first step of autophagy, begins with the cell’s activation of the Unc51-like kinase 1 (ULK1) complex which signals the cell to begin formation of the autophagosome. Elongation and maturation remain under the control of the microtubule-associated protein 1 light chain 3 (LC3) and Atg12 system. During these steps, double-membrane vesicles and autophagosomes will form^3^. Autophagosomes engulf cellular components and debris. Finally, the autophagosomes fuse with lysosomes, through the formation of an autolysosome intermediary, which results in digestion of their contents^4^.

Autophagy’s role in the pathogenesis of human diseases appears contextual with responses varying by disease type^5^. Cancer studies have shown that under certain circumstances autophagy can be tumor suppressive or tumor promoting^6^. However, the exact processes by which autophagy can assume either of these roles remain under investigation. One overriding theory is that catabolism acting through autophagy leads to cell survival, whereas cellular imbalances in autophagy can lead to cell death^7^. In some cases, cancer cells have been shown to be more autophagy dependent than normal cells, likely due to microenvironment deficiencies and high metabolic demands^8^. Although further understanding of the context-dependent biological functions and regulation of autophagy is needed, modulation of this process is an attractive approach for future cancer drug discovery^1,6]^.

The clinically approved antimalaria drug chloroquine (CQ) and its derivatives such as hydroxychloroquine (HCQ) are the most utilized autophagy inhibitors to date^6,9^. CQ and HCQ are thought to block late-stage autophagic flux by accumulating inside endosomes and lysosomes, leading to deacidification which in turn impairs enzymatic function^10^. They are not ideal inhibitors because they lack specificity, and as a result, they impact the overall lysosomal function^1,11^. In addition, studies have suggested other potential mechanisms underlying CQ’s cytotoxicity in cancer, including its ability to promote DNA damage at high doses^12^ and to enhance anti-angiogenic effects^13^. Furthermore, there has been an inconsistency in tumor responses to autophagy inhibition in clinical trials due to the ability of the drug to penetrate evenly through a tumor and potential toxicity when used in combination with other chemotherapeutic agents^6^.

Despite potential limitations to CQ and HCQ, there is evidence from our group and others for the efficacy of this approach for tumors that rely on autophagy for proliferation and survival. Recent studies have suggested that tumors harboring mutations in RAS and BRAF develop an “addiction” to autophagy for maintaining cellular homeostasis. Therefore, blocking autophagy causes enhanced cell death^14–18^. Studies by Guo et al. demonstrated the profound effect of genetic inhibition of autophagy in lung tumors harboring the mutant RAS^19^. Similar effects were seen in BRAF^V600E^-driven lung tumors^20^. We have shown that BRAF^V600E^ glioma cells demonstrated more dependency on autophagy for survival compared with BRAF wild-type cells. BRAF mutant cancers may be particularly sensitive to autophagy inhibition when combined with BRAF inhibition (BRAFi) as autophagy can be induced as a survival mechanism, potentially limiting drug efficacy^17,21^. In addition, we have demonstrated that autophagy inhibition overcomes the resistance in BRAFi-resistant tumor cells in vitro and in patients^18^. Most recently, autophagy inhibition has also been shown to be a potential target in RAS-activated pancreatic cancer^14,16^.

Due to concerns over nontarget effects of CQ and HCQ, development and characterization of more specific small-molecule inhibitors targeting alternative components of the autophagy pathway is ongoing^22^. ULK1, the only serine/threonine kinase involved in the autophagy pathway, represents a potential target^23^. ULK1 is the mammalian ortholog of yeast ATG1 and is regulated by cellular amino acid and energy status via both mTORC1 (the mechanistic target of rapamycin-1) and AMPK (AMP-activated protein kinase) kinases. mTORC1 signaling coordinates energy and nutrient availability with cell growth and metabolism. Under nutrient-rich conditions, it phosphorylates ULK1 and ATG13 (a member of the ULK1 complex), reducing ULK1 kinase activity and in turn inhibiting autophagy. AMPK is active in low-energy states, by inactivating mTORC1 resulting in its dissociation with ULK1 and leading to autophagy activation. There are various ATP-competitive inhibitors against ULK1 kinase^24^, including the selective SBI-0206965 (SBI)^23^. SBI has been shown to effectively inhibit autophagy in non-small-cell lung cancer cells^23,25^ as well as synergize with mTOR inhibition^23^ and other standard chemotherapies^25^.

VPS34 (vacuolar protein sorting 34), a class III PI3K, also plays a critical early role in the development of autophagosomes, making it an attractive target. It belongs to the Beclin1 complex that produces PI3 phosphate, a lipid important for autophagosome membrane formation and vesicle trafficking. Multiple VPS34 inhibitors have been investigated^26–28^. Inhibition of VPS34 with SAR405 in combination with mTOR inhibition has been shown to reduce cell growth in renal tumor cells^28^. VPS34-IN1 is another highly potent and selective VPS34 inhibitor with its potential for use in cancer therapy^26^.

As we have demonstrated that late-stage autophagy inhibition is effective in improving treatment in BRAF^V600E^ autophagy- dependent tumors, we sought to determine if early-stage autophagy inhibition can also be effective in this tumor population, leading to increased clinical options for therapy. This study evaluated early-stage inhibition through ULK1 or VPS34 as a potential therapy option in autophagy-dependent central nervous system (CNS) tumors.

## Materials and methods

### Study design

Experiments were designed to evaluate the hypothesis that early-stage autophagy inhibition will effectively inhibit cell growth and survival in autophagy-dependent CNS tumor cells. This was evaluated in vitro in cell lines. To ensure a complete evaluation of the effects on cell growth and death, both long- and short-term growth assays were utilized. The specificity to the autophagy pathway was evaluated with genetic inhibition studies. The final endpoints were defined prior to the start of each experiment. All in vitro experiments were completed with two to three biological replicates and where possible with triplicate technical replicates. Details on replicates and statistical analysis are indicated in the figure legends.

### Statistics

Statistical comparisons were completed by using an ordinary two-way ANOVA, one-way ANOVA, and Dunnett’s multiple comparison tests (GraphPad Prism 7.04, RRID: SCR_002798) as indicated in the figure legends. A *P*-value of <0.05 was considered statistically significant. The data shown are mean ± standard error of the mean (SEM) except where indicated.

### Cell culture and reagents

The AM38 (PRID:CVCL_1070) cell line was purchased from the Japan Health Sciences Foundation Health Science Research Resources Bank (Osaka, Japan). AM38 cells were derived from a 36-year-old glioblastoma patient with standard glioblastoma markers, including GFAP and S-100-positive histology; genetic analysis revealed a BRAF^V600E^ mutation. The MAF794 is a primary patient cell line collected and established in accordance with local and federal human research protection guidelines and institutional review board regulations (COMIRB 95–500). The MAF794 cell line was derived from a 6-year-old patient diagnosed with a right parietal mass with two distinct components, a low-grade ganglioglioma with rhabdoid morphology. Mutational analysis demonstrated SMARCB1 loss in addition to a BRAF^V600E^ mutation^29^. Cell line authentication was performed by using short tandem-repeat profiling and then comparing that data with the known cell line DNA profiles. Mycoplasma contamination testing was performed by using a Lonza MycoAlert Mycoplasma Detection Kit (Lonza Ltd., Switzerland).

AM38 cells were cultured in MEM supplemented with 20% fetal bovine serum (FBS) (#S11150, Atlanta Biologicals, Flowery Branch, GA) containing 1% Penicillin/streptomycin (#15070063, Thermo Fisher Scientific, Waltham, MA). MAF794 cells were cultured in OptiMEM supplemented with 15% FBS (#S11150, Atlanta Biologicals, Flowery Branch, GA) containing 1% Penicillin/streptomycin (#15070063, Thermo Fisher Scientific, Waltham, MA). Acidic media was prepared by the addition of 1 N acidic acid to the standard media. The media was then allowed to equilibrate at 4 °C overnight. Cells were plated in standard media buffered at normal pH. The next day, the media was replaced by acidic media followed by treatments as indicated.

Cell lines were maintained at 37 °C in a humidified incubator in an atmosphere of 5% CO_2_ according to the cell line-specific growth requirements. BRAFi-resistant cell lines were generated through chronic exposure to increasing doses of vemurafenib as previously described^17^.

Vemurafenib (#V-2800) and chloroquine (#193919) were purchased from LC Laboratories (Woburn, MA) and MP Biomedicals (Santa Ana, CA) respectively. SBI-0206965 (#SML-1540) was purchased from Sigma-Aldrich (St. Louis, MO) and VPS34-IN1 (#S7980) was obtained from Sellekchem (Houston, TX).

### In vitro viability assays

For short-term viability assays, cells were seeded in 96-well plates at a density of 1000 cells/well followed by the treatment as indicated and incubated at 37 °C in 5% CO_2_. After 5 days of treatment, cell viability was determined by using CellTiter-Glo® Luminescent Cell Viability Assay according to the manufacturer’s protocol (#G7572, Promega Corporation, Madison, WI).

Apoptotic cell death was measured by using Guava Nexin assay (Luminex Corporations). This assay uses 7-AAD as an indicator of the membrane structural integrity and Annexin V-PE for the detection of phosphatidylserine present on the external membrane of apoptotic cells. Cells were plated at a density of 50,000 cells/well in six-well plates followed by 48-h treatment as indicated. Cells were harvested, washed with 1×PBS, and stained with Guava Nexin reagent for 20 min at room temperature in the dark. Apoptotic cells in each condition were then measured by using the Guava EasyCyte flow cytometer (Luminex Corporations). The amount of early apoptotic (Annexin V+ and 7-AAD–) and late apoptotic dead cells (Annexin V+ and 7-AAD+) in treated cells were compared with untreated controls.

Long-term survival was assessed by using a colony-formation assay. Cells were plated into 12-well plates at a density of 200–400 cells/well in accordance with the optimal growth condition of each cell line and incubated overnight at 37 °C in 5% CO_2_. Cells were treated with a IC_50_ dose of drugs as previously described^17^ and incubated for 10–14 days. Colonies were monitored and provided with fresh media with or without drugs every 3 days. When the control wells reached 80–85% confluence, the cells were fixed, stained with 0.4% crystal violet, and quantified by solubilization in 33% (v/v) acetic acid with A540 absorbance assessed.

### IncuCyte growth measurement assay

Cells transduced with IncuCyte® NucLight Red Lentivirus Reagent (Cat. No. 4476, Essen BioScience Inc., Ann Arbor, MI) were seeded in 96-well plates (Costar, Corning, NY) at a density of 1000 cells/well and incubated inside an IncuCyte Zoom (BioScience Inc., Ann Arbor, MI) overnight. Cells were then treated as described and monitored. Select experiments incorporated the CellEvent caspase-3/7 Green reagent to assess for apoptosis. Four images in separate regions of each well were captured at 4-h intervals by using a 10× objective. To create and analyze the growth curves, fluorescent nuclei or green object count were measured over time and presented as percent growth, percent confluence, or a measure of area under the curve (AUC).

### Western blot analysis and antibodies

Cell lysates were harvested after treatments and time points indicated by using RIPA buffer (Sigma, St. Louis, MO) with protease inhibitor cocktails (Roche, Indianapolis, IN) following treatments and time points as indicated. Samples were boiled for 10 min at 95 °C, and they were resolved by SDS-PAGE. Membranes were blocked with 5% dry nonfat milk in TBS-Tween for 1 h at room temperature and probed with primary antibodies at the manufacturer’s recommended concentrations.

The primary antibodies used were PI3 kinase class III/VPS34 (#3811S, RRID: AB_2062856); Total ULK1 (#4773S, RRID: AB_2288252); Phospho-p44/42 MAP kinase (#9101S, RRID: AB_331646); p44/42 MAP kinase (Erk1/2) (#9102, RRID: AB: 330744); LC3 (#NB100–2220, RRID: AB_10003146) (Novus Biologicals, Littleton, CO); p62/SQSTM1 (#H00008878-M01, RRID: AB_437085) (Abnova). Anti-β-actin (#12262, RRID: AB_2566811) (Cell Signaling, Danvers, MA) was used as the protein-loading control. Secondary antibodies were purchased from Cell Signaling Technology including anti-rabbit IgG (#7074S, RRID: AB_2099233) and anti-mouse IgG (#7076S, RRID: AB_330924). The results of western blots were assessed by comparing the intensity of bands by using Image J (RRID: SCR_003070).

### Flow-cytometry analysis

Cells constitutively expressing mCherry-GFP-LC3^17^ were plated at 2.4 × 10^5^ in 6-cm dishes and incubated overnight at 37 °C in 5% CO_2_. Cells were then exposed to both the standard media and Earl’s Balanced Salt Solution (EBSS) starvation media (#2888, Sigma, St. Louis, MO) in the absence or the presence of the drug as indicated. For genetic inhibition experiments, cells were plated at 1.5 × 10^5^ per well in six-well plates for 48 h following transduction. The following day, cells were exposed to either the standard media or EBSS as indicated prior to flow analysis. Data were acquired on a Galios561 (Beckman Coulter, RRID: SCR_008940, Fort Collins, CO) and analyzed by using FlowJo V10.0.8 (RRID: SCR_008520). The mCherry signal was excited by a 561-nm laser and acquired in the FL3 filter. The GFP signal was excited by a 488-nm laser and acquired in the FL1 filter. Autophagic flux was determined by the ratio change in the median fluorescence intensity of mCherry:GFP.

### Live-cell fluorescence imaging

Cells were plated in six-well plates overnight. Twenty-four hours following exposure to standard media and EBSS in the presence or absence of SBI or VPS34-IN1, the status of autophagy was analyzed by fluorescence microscopy. Representative images of AM38 P and AM38 R cells were captured by using a 20× lens on a Keyence BZ-X710 microscope. Scale bars: 50 µm. AM38 P cells are shown with GFP, mCherry, and merged images to demonstrate the dual mch:GFP:LC3 signal. AM38 P and AM38 R merged images are also shown to demonstrate the loss of the GFP signal in EBSS-treated cells that is rescued in the presence of SBI or VPS34-IN1.

### shRNA transfection

A pLKO.1-puro lentiviral vector from the RNAi Consortium (TRC; Sigma-Aldrich) was utilized with small-hairpin RNAs (shRNAs). TRC numbers for shRNAs used were as follows: ULK1 #1 (#199801), ULK1 #2 (#195477), VPS34 #1 (#196840), and VPS34 #2 (#196290), the nontarget (SHC016). Cells were transduced with a lentivirus by using 8 µg/µl polybrene and harvested 3 days following transduction. The level of target gene knockdown and its effect on autophagic flux was determined via both western blotting and flow cytometry.

### Synergy measurements

The combination index was calculated by the Chou–Talalay equation, which takes into account both the potency (IC_50_) and shape of the dose-effect curve (the *M*-value)^30^. Combination index values <1, =1, and >1 indicate synergism, the additive effect, and antagonism, respectively.

## Results

### Pharmacologic inhibition of early-stage autophagy diminishes CNS tumor cell viability in vitro

**Fig. 1:**
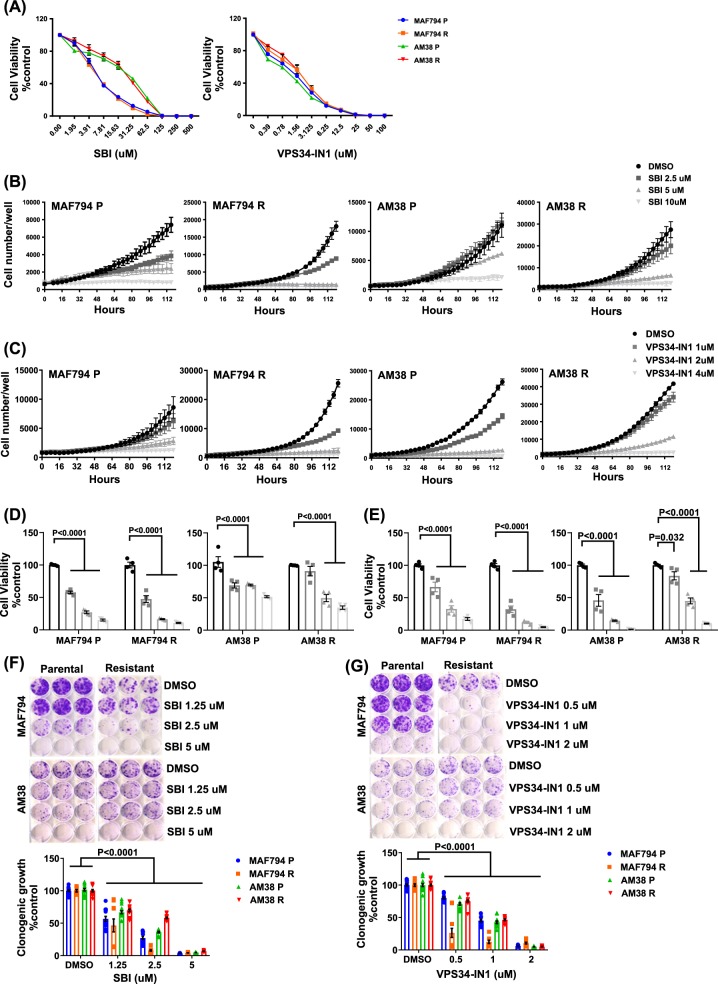
Parental and resistant BRAF^V600E^ brain tumor cell lines demonstrate sensitivity toward early-stage autophagy inhibition. **a** Effect of SBI or VPS34-IN1 on short-term viability in MAF794 and AM38 parental (P) and resistant (R) cells treated with increasing doses of SBI or VPS34-IN1 for 5 days. Viability was determined by using the CellTiter Glo assay. **b**, **c** Growth curves of MAF794 and AM38 P and R cells following SBI (**b**) or VPS34-IN1 (**c**) treatment. Cell number per well was obtained over time by using Incucyte Zoom (Essen Bioscience). **d**, **e** Percent cell viability compared with DMSO control measured by CellTiter Glo assay following a 5-day treatment of SBI (**d**) or VPS34-IN1 (**e**). **f**, **g** Representative long-term clonogenic assays and quantified collated data of cells treated with SBI (**f**) or VPS34-IN1 (**g**) as indicated. Dunnett’s multiple comparisons; mean ± s.e.m., *n* = 2. *p < 0.05

To assess the pharmacologic sensitivity of parental and resistant BRAF^V600E^ (MAF794 and AM38) cell lines toward ULK1 and VPS34 inhibition, cells were treated with SBI or VPS34-IN1 and their viability was measured. Following continuous exposure of cells to increasing doses of the drug for 5 days, viability was reduced in a dose-dependent manner in all cell lines (Fig. 1a). IC_50_ values for each drug (Table 1) indicated a lower IC_50_ for VPS34-IN1 compared with SBI. AM38 cells (parental and resistant) had a significantly higher IC_50_ for SBI when used as monotherapy than MAF794 cells.

**Table 1:** IC_50_ values for SBI and VPS34-IN1 in BRAF^V600E^ parental and resistant cells

Cell lines		
	SBIuM	VPS34-IN1uM
MAF794 P	3.59	1.98
MAF794 R	3.85	2.30
AM38 P	37.13	1.62
AM38 R	27.91	2.13

Values were calculated for inhibition of growth of BRAF^V600E^ cell lines by using GraphPad Prism Software 7.04 (GraphPad Software Inc.) from dose–response studies (Fig. 1)

The dose–response relationship was further evaluated. For short-term growth and viability analysis, cells were treated with doses surrounding the IC_50_ and monitored by using IncucyteZOOM. Real-time measurements of cell growth over time (Fig. 1b, c) together with endpoint cell viability measurements (Fig. 1d, e) demonstrated a dose-dependent decrease in cell survival and growth for both drugs in all cell lines tested. In addition, clonogenic long-term growth assays indicated a significant dose-dependent sensitivity in all cell lines toward both SBI and VPS34-IN1 (Fig. 1f, g).

Taken together, our data suggest that both early-stage autophagy inhibitor candidates, SBI and VPS34-IN1, affect the survival of BRAF^V600E^ CNS tumor cells during both short- and long-term in vitro growth analyses, irrespective of their BRAFi sensitivity.

### Pharmacologic inhibition of ULK1 and VPS34 reduces autophagic flux

**Fig. 2:**
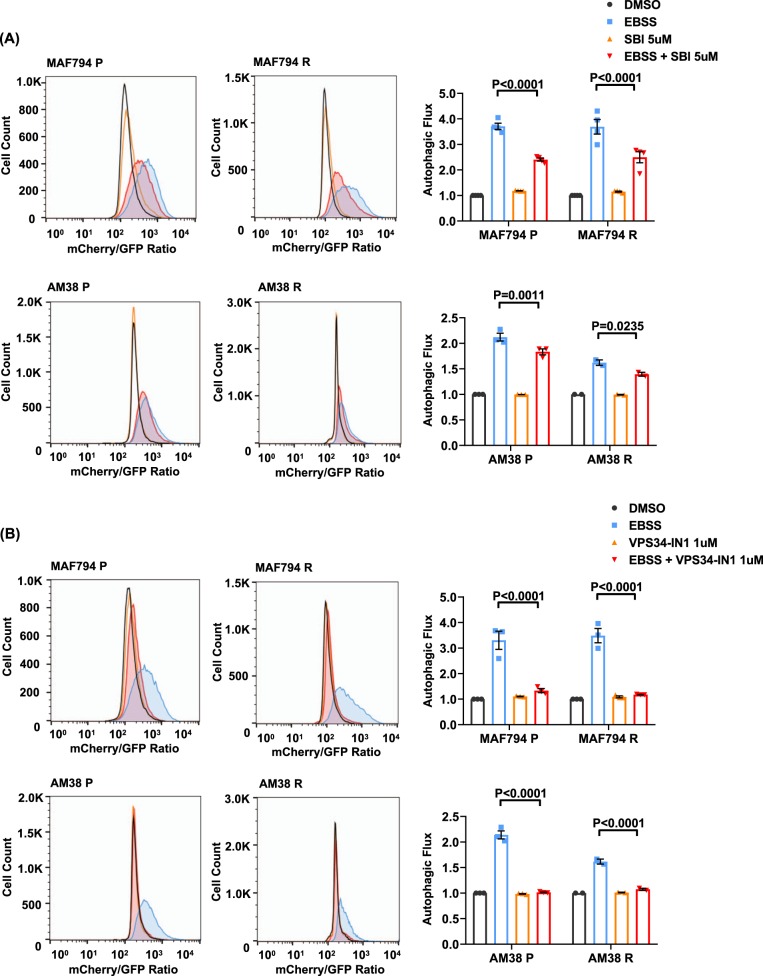
Autophagic flux is reduced following pharmacological inhibition of early-stage autophagy. **a**, **b** Representative histograms and quantifications (Dunnett’s multiple comparisons; mean ± s.e.m., *n* = 3. *p < 0.05) of basal and induced autophagy in parental and resistant MAF794 and AM38 cells. mCh-GFP-LC3 cells were exposed to standard and serum starvation media (EBSS) for 24 h in the presence or absence of SBI (**a**) or VPS34-IN1 (**b**) and analyzed by flow cytometry. Autophagic flux was determined by the ratio change in the median fluorescence intensity of mCherry:GFP and normalized to DMSO control

**Fig. 3:**
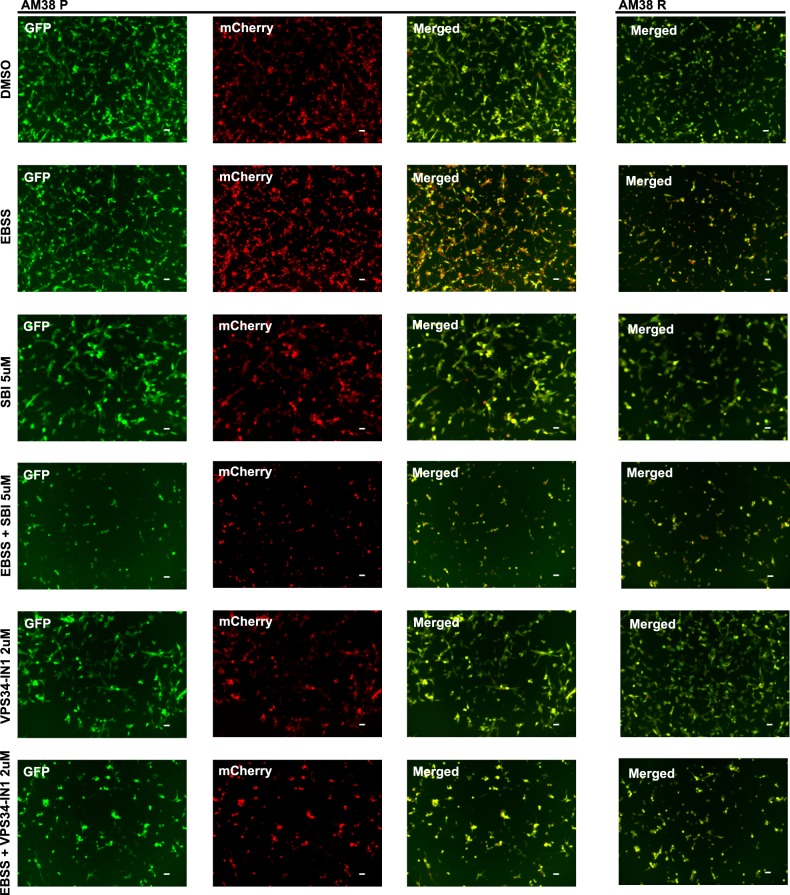
Live-cell fluorescence imaging of mch-GFP-LC3-tagged AM38 P and AM38 R cells demonstrates the rescue of the GFP signal with early-stage inhibition in the presence of serum starvation. Cells were plated in six-well plates overnight. Twenty-four hours following exposure to standard media and EBSS in the presence or absence of SBI or VPS34-IN1, the status of autophagy was analyzed by fluorescence microscopy. Representative images of AM38 P and AM38 R cells were captured by using a 20× lens. Scale bars: 50 µm. AM38 P cells are shown with GFP, mCherry, and merged images to demonstrate the dual mch:GFP:LC3 signal. AM38 P and AM38 R merged images are also shown to demonstrate the loss of the GFP signal in EBSS-treated cells that is rescued in the presence of SBI or VPS34-IN1

To evaluate if the observed effects of SBI and VPS34-IN1 in reducing tumor cell survivability are associated with a concomitant decrease in autophagy, we evaluated basal (nutrient-rich) and induced (serum-starved) autophagic flux by flow cytometry and fluorescent microscopy. In cells with a tandem mCh-GFP-LC3, the GFP signal is quenched by the acidic environment of the autophagosome following fusion with the lysosome. Comparison of the ratio of the median fluorescence intensity of mCh:GFP allows for a quantitative measure of autophagy. Under nutrient-rich conditions, there was no measurable difference in autophagic flux following the addition of either SBI or VPS34-IN1 (Fig. 2a, b). In comparison, serum-starved cells demonstrated a significant increase in autophagy which was reduced following the addition of either SBI or VPS34-IN1 (Fig. 2a, b). The loss of the GFP signal can be seen in AM38P and AM38R cells (Fig. 3) treated with EBSS. Merged images show quenching of GFP in EBSS-treated cells. Rescue of the GFP signal that correlates to quantitative flow data can be seen in cells co-treated with EBSS and either SBI or VPS31-IN1 indicating inhibition of autophagy. Of note, VPS34-IN1 was more effective in reducing the induced autophagic flux compared with SBI in all cell lines.

Additional evaluation of basal flux was performed by western blot (Supplementary Fig. 1). Non-stressed cells were treated with or without Bafilomycin-A (Baf), an inhibitor of lysosomal fusion to autophagosomes, and accumulation of LC3II and p62 was evaluated. With SBI, there is a decreased accumulation of LC3II compared with control in all cell lines except MAF794P, indicating decreased basal autophagy (Supplementary Fig. 1A). Treatment with VPS34-IN1 also resulted in a decrease in LC3II accumulation (Supplementary Fig. 1B). Rescue of p62 degradation was also decreased following treatment with SBI or VPS34-IN1 (Supplementary Fig. 1A-B).

Collectively, these data demonstrated that pharmacologic inhibition of either ULK1 or VPS34-IN1 successfully inhibits autophagic flux in our BRAF^V600E^ CNS tumor cells. These inhibitors are effective in both parental and BRAFi-resistant cells.

### Pharmacologic early-stage autophagy inhibition improves the effectiveness of BRAFi in resistant cell lines

**Fig. 4:**
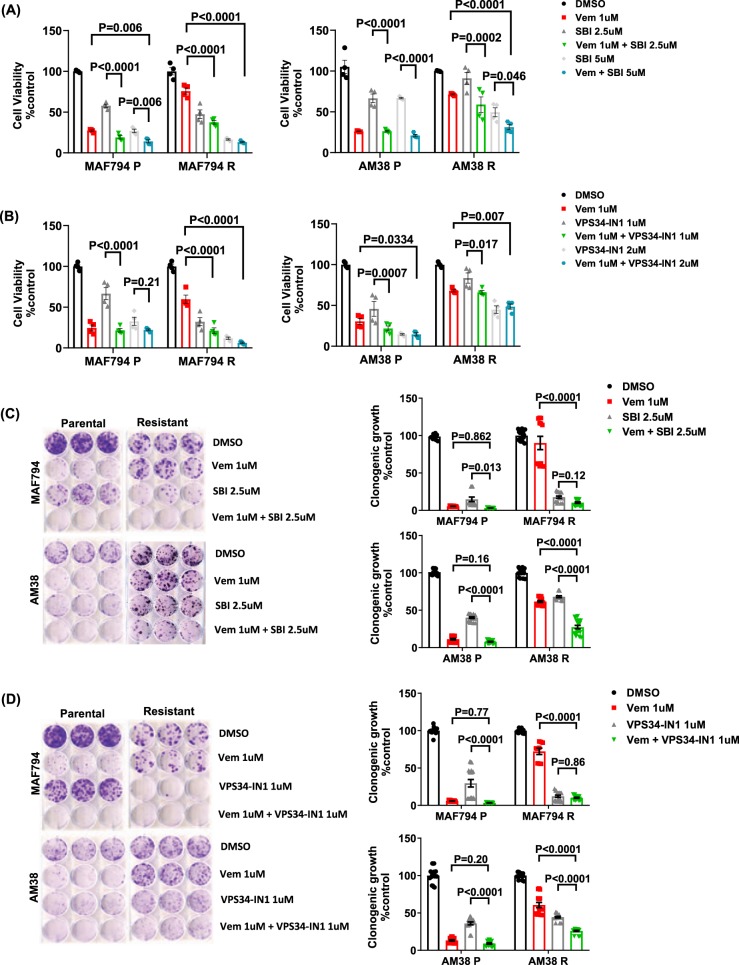
Pharmacologic early-stage autophagy inhibition improves the effectiveness of BRAFi in BRAF^V600E^ CNS tumor cells. **a**, **b** Percentage of cell viability as measured by CellTiter Glo (compared with control DMSO) following a 5-day exposure to SBI (**a**) or VPS34-IN1 (**b**) in the presence or absence of BRAFi. **c**, **d** Representative long-term clonogenic assays with quantified collated data following treatment of cells with SBI (**c**) or VPS34-IN1 (**d**) in the presence or absence of BRAFi as indicated. Dunnett’s multiple comparisons; mean ± s.e.m. (*n* = 2). *p < 0.05

To assess the combined effect of early-stage autophagy inhibition with BRAFi, cells were exposed to SBI or VPS34-IN1 in the presence or absence of vemurafenib for 5 days (Fig. 4). Viability was quantified by using theCellTiter Glo assay. MAF794 parental and resistant cells demonstrated a significant reduction in cell survival following SBI treatment (Fig. 4a). MAF794R cells showed an expected decrease in the sensitivity to BRAFi with vemurafenib, which was significantly improved with the addition of SBI. As previously demonstrated in Fig. 1a, AM38 cell lines were less responsive to SBI alone. AM38R BRAFi-resistant cells showed the expected decreased sensitivity to vemurafenib, and this sensitivity was significantly improved with the addition of SBI at 5 µM (Fig. 4a).

We additionally assessed the effect of VPS34-IN1 in the presence or absence of BRAFi. Similar to cells treated with SBI, MAF794 cells were sensitive to VPS34-IN1 treatment alone and MAF794R sensitivity to vemurafenib was significantly improved with the addition of VPS34-IN1 (Fig. 4b). AM38 cells were similarly sensitive to VPS34 inhibition as shown in Fig. 1a. AM38R cells have the expected decreased sensitivity to vemurafenib, but there is no evidence of synergy with the addition of VPS34-IN1 in this assay (Fig. 4b).

To more accurately assess the efficacy of combining BRAFi and ULK1 or VPS34 pharmacologic inhibition, viability was measured following 5 days of treatments, and the combination index (CI) values were calculated by the Chou–Talalay method^30^ (Table 2). In the presence of BRAFi, all cell lines exhibited a synergistic response when treated with SBI irrespective of their BRAFi sensitivity (Table 2). However, a combination of VPS34-IN1 and BRAFi only appeared to increase drug sensitivity in MAF794 parental and resistant cells. AM38 cells demonstrated an antagonistic response to the combination therapy (Table 2). These results were consistent with the combination data previously described in Fig. 4a, b.

**Table 2:**
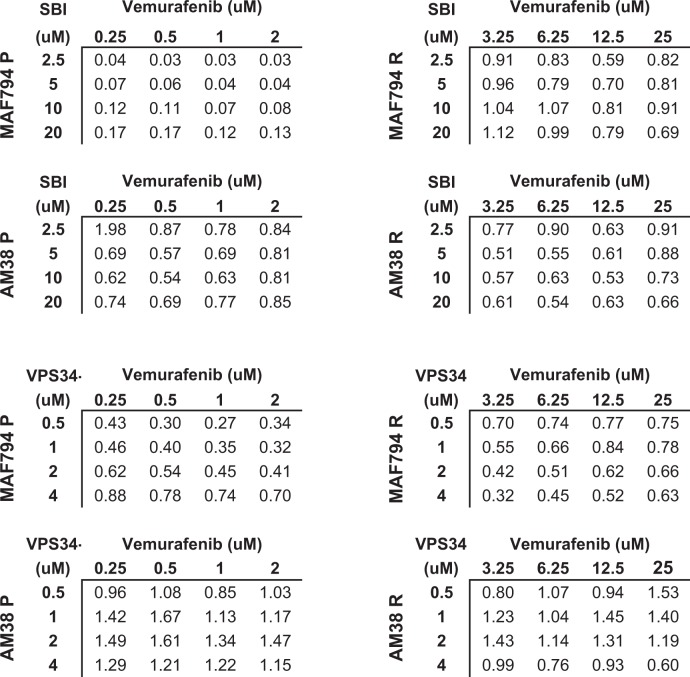
Combination index (CI) for SBI or VPS34-IN1 in addition to BRAFi in BRAF^V600E^ parental and resistant cells

Parental and resistant cells were treated with increasing doses of SBI or VPS34-IN1 and vemurafenib for 5 days. CellTiter Glo assays were performed to assess cell viability. The Chou–Talalay equation was used to calculate IC_50_ and determine synergism. Combination index (CI) values <1, =1, and >1 indicate synergy, additives, and antagonism, respectively (*n* = 2)

To test if longer therapy would result in improved response to a combination of BRAF and early autophagy inhibition, we performed combination therapy clonogenic assays. In MAF794 and AM38 parental cells, treatment with vemurafenib was highly effective at decreasing cell survival, making additional benefits of SBI or VPS34-IN1 difficult to assess (Fig. 4c, d). In comparison, the addition of either SBI or VPS34-IN1 to BRAFi in the resistant cells resulted in a significant decrease in cell survival with combination therapy (Fig. 4c, d).

### Pharmacologic early-stage autophagy inhibition results in caspase activation and apoptotic cell death in BRAF^V600E^ CNS tumor cells

To assess if the noted decrease in cell viability was due to cell death or decreased proliferation, we analyzed the presence of Annexin V-positive cells following 48 h of treatment (Supplementary Fig. 2). The amount of Annexin V-positive cells indicating apoptotic cell death was significantly increased in cells treated with the early-stage autophagy inhibitors compared with control. In most conditions tested, there was also a significantly higher percentage of apoptotic cell death detected in combination therapy over BRAFi single-drug therapy. AM38R cells did not show a significant increase in Annexin V-positive cells following treatment with VPS34-IN1 alone or in combination with BRAFi (Supplementary Fig. 2B). This would suggest that the moderate differences seen among treatment groups by CellTiter Glo could represent changes in proliferation as opposed to cell death.

To further analyze the role of apoptosis in the treatment of these tumors with early-stage autophagy inhibitors, we monitored treatments of cells in the presence of CellEvent caspase-3/7 (Supplementary Fig. 3). Consistent with the CellTiter Glo and Annexin V data, cells treated with SBI or VPS34-IN1 demonstrated significantly increased caspase activation over control or BRAFi alone in all treatments with the exception of AM38R cells treated with VPS34-IN1. Similar to the Annexin V data, treatment with VPS34-IN1 alone or in combination with AM38R cells had a much lower caspase activation, again suggesting that the results seen with these cells could be related more to changes in proliferation.

### Nutrient limitation but not pH changes affect the response to pharmacologic early-stage autophagy inhibition

We assessed the impact of nutrient limitation and pH changes on tumor cell response seen above as it has been shown that both can cause changes in the response to autophagy inhibition^23,24,31^. Cells were exposed to SBI or VPS34-IN1 in the presence or absence of BRAFi under both nutrient-rich and serum-starved (nutrient-poor) conditions (75% reduced-serum media). As shown in Supplementary Fig. 4A-B, under nutrient-poor conditions, cell viability was reduced in MAF794 parental and resistant cells following SBI or VPS34-IN1 and BRAFi treatments similar to nutrient-rich conditions. AM38 parental and resistant cells demonstrated a significant reduction in cell viability under nutrient-poor conditions that was further reduced following both autophagy and BRAFi. It is interesting to note that VPS34-IN1 had a greater impact on both AM38 parental and resistant cell viability compared with SBI which was further enhanced by the addition of BRAFi (Supplementary Fig. 4A-B). The combination effect was more pronounced in AM38R cells. Overall, these data suggest that nutrient availability may play a role in CNS tumor cell response to early-stage autophagy inhibition.

It has been reported that use of CQ is not able to block autophagy in cells cultured at an acidic pH^31^ that mimics the tumor microenvironment of the center of solid tumors which can be hypoxic and acidic. To assess this in our cells, MAF794P (Supplementary Fig. 5A-B) and MAF794R (Supplementary Fig. 6A-B) cells were cultured under normal (pH = 7.7) and acidic (pH = 6.8) conditions and treated as above. There was no significant difference in response under normal or acidic conditions. Evaluation of autophagy by western blot of LC3II (Supplementary Fig. 5C and 6C) also did not demonstrate a significant difference of LC3II accumulation in normal or acidic pH.

Collectively, these data indicated that both SBI and VPS34-IN1 are effective at decreasing cell survival as monotherapy, and that the addition of these drugs significantly improves the response of BRAFi-resistant cells to vemurafenib. This effect is best demonstrated with a long-term growth assay which would be similar to the continuous therapy provided to patients clinically.

### Genetic inhibition of early-stage autophagy overcomes resistance to and improves sensitivity toward BRAFi treatment

**Fig. 5:**
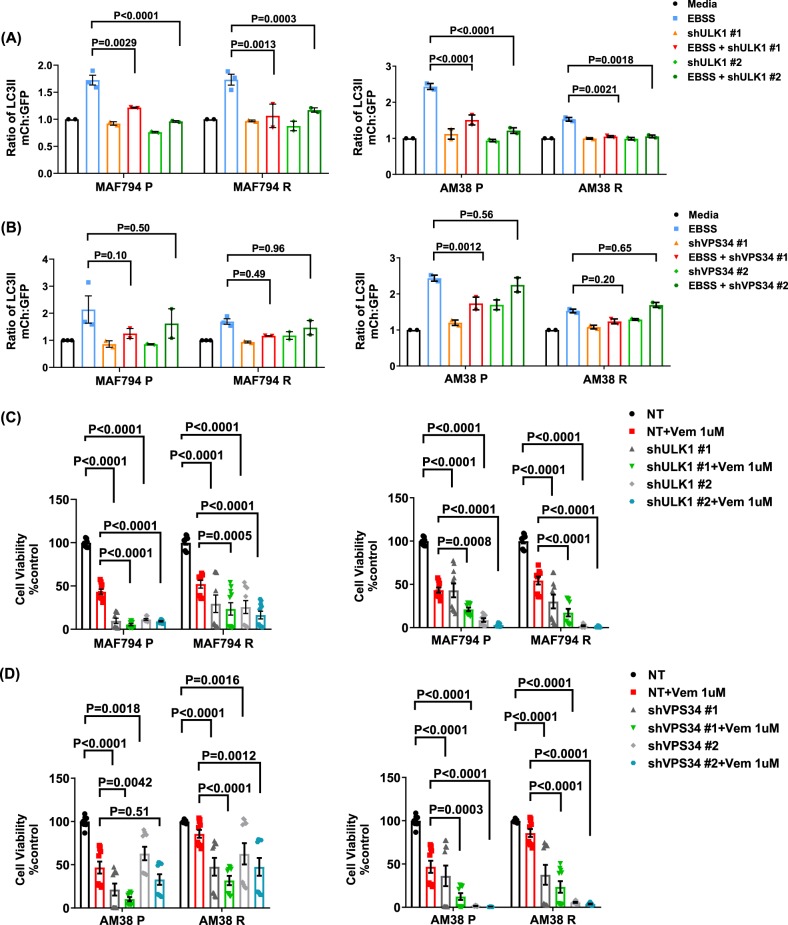
Genetic early-stage autophagy inhibition improves sensitivity toward BRAFi. **a**, **b** Quantifications of basal and induced autophagy in MAF794 and AM38 parental and resistant cells following autophagy inhibition through RNAi against ULK1 (**a**) or VPS34 (**b**) compared with non-targeting (NT) RNAi. Autophagic flux was determined as previously described. **c**, **d** Percent cell viability demonstrating the effectiveness of autophagy inhibition through RNAi against ULK1 (**c**) or VPS34 (**d**) compared with NT RNAi in the presence or absence of BRAFi. Percent cell viability was measured by CellTiter Glo assay following 5-day exposure to vemurafenib with or without RNAi against ULK1 or VPS34. Dunnett’s multiple comparisons; mean ± s.e.m. (*n* = 2). *p < 0.05

To evaluate whether the observed pharmacologic effects of SBI or VPS34-IN1 were specifically related to the inhibition of the autophagy pathway, we genetically inhibited ULK1 and VPS34 and evaluated the changes in the autophagic flux by flow cytometry, survival by CellTiter Glo, and synergy with BRAFi. Acute RNAi of ULK1 or VPS34 (two shRNAs per target) was performed in cells expressing mCh-GFP-LC3. Confirmation of protein knockdown was assessed by western blot analysis (Supplementary Fig. 7A-B). In standard media, knockdown of ULK1 or VPS34 did not significantly affect basal autophagy (Fig. 5a, b). All cells demonstrated a significant increase in autophagy in EBSS starvation media (Fig. 5a, b and Supplementary Fig. 8A-B). Of note, AM38R cells had a smaller increase in the autophagic flux than that seen in other cell lines. Starvation-induced autophagy was significantly reduced in the presence of ULK1 knockdown in all cells (Fig. 5a and Supplementary Fig. 8A). VPS34 knockdown had a lesser effect in reducing autophagy under starvation (Fig. 5b and Supplementary Fig. 8B). Together, our results demonstrated the inhibition of an induced autophagic flux following the genetic inhibition of both ULK1 and VPS34 (Fig. 5a, b and Supplementary Fig. 8A-B).

To evaluate the effect of genetic inhibition on growth and viability, cells were monitored via IncuCyte in the presence or absence of acute RNA interference (RNAi) against ULK1 and VPS34 with or without BRAFi (Fig. 5c, d and Supplementary Fig. 9A-B). The rate of cell growth (Supplementary Fig. 9) and viability (Fig. 5c, d) was significantly reduced following the genetic inhibition of ULK1 or VPS34 in all cells. As expected, MAF794P cells demonstrated a significant reduction in cell viability following vemurafenib treatment. This was further reduced in the presence of RNAi. MAF794R cells exhibited lesser sensitivity to vemurafenib treatment but the resistance was overcome following RNAi therapy. Similar results were obtained in AM38 cells. In both parental and resistant MAF794 and AM38 cells, BRAFi in combination with ULK1 or VPS34 knockdowns resulted in a significant reduction in cell viability when compared with either treatment alone (Fig. 5c, d). Together, these data suggest that the reduction in tumor growth and viability in response to SBI and VPS34-IN1 is related to the inhibition of the autophagy pathway. Importantly, combination therapy in resistant cells significantly improved the response to BRAFi.

## Discussion

Although considerable progress has been made in developing novel molecular targeted therapies, many challenges still remain in bringing safe and effective therapeutics to patients. In particular, the eventual development of drug resistance continues to remain a constant challenge^32^. Recent studies support the idea of autophagy facilitating cancer cell survival and resistance to chemotherapeutic drugs. Therefore, autophagy inhibition may improve tumor cell response to treatments^33^. Recognition and diagnosis of CNS tumors with BRAF^V600E^ mutations are increasing with the expanded availability of tumor genetic analysis, particularly in subsets of CNS tumors including gangliogliomas, pleomorphic xanthoastrocytomas, and epithelioid glioblastomas^34,35^. The identification of BRAF^V600E^ is important as there are no histopathological differences between tumors with and without the mutation. More importantly, the presence of BRAF^V600E^ has been associated with a more aggressive phenotype^36^. The use of targeted therapy with BRAFi is becoming an important component of CNS tumor therapy^37^.

We previously reported that BRAF^V600E^ CNS tumors demonstrated high rates of induced autophagy and increased the sensitivity toward BRAFi compared with wild-type tumors^17^. We also demonstrated the beneficial effects of late-stage autophagy inhibition in overcoming the resistance to BRAFi in vitro, ex vivo, and most importantly in patients^18^. These results suggest a role in autophagy inhibition in BRAF^V600E^ CNS tumors.

Additional studies have shown that autophagy inhibition at early or late stages can have differential effects on tumor cell death. For instance, in prostate cancer, we showed that early-stage autophagy inhibition reduced tumor cell death, whereas inhibition of autophagy at a later stage resulted in an increase in cell death^38,39^. Therefore, understanding which inhibitory process to target is essential in designing the most effective therapeutic regimens for patients, particularly BRAF^V600E^ CNS tumors that are autophagy dependent.

In the current study, we provide the results demonstrating the effectiveness of pharmacologic and genetic early-stage autophagy inhibition in both BRAFi-sensitive and resistant CNS tumor cells through a combination of flow cytometry, western blotting, and cell viability assays. We evaluated the inhibition of ULK1 and VPS34 given their potential for future clinical use and increased specificity for autophagy inhibition compared with CQ or HCQ. Following ULK1 inhibition, both pharmacologically by SBI and genetically through RNAi, our results indicated diminished growth and viability in all cell lines irrespective of their BRAFi sensitivity. Interestingly, resistant cells demonstrated even a more pronounced response to autophagy inhibition and BRAFi combination therapy. These results are consistent with previous studies performed in non-small-cell lung cancer (NSCLC)^25^. Tang et al. demonstrated that both pharmacological and genetic inhibition of ULK1 in NSCLC resulted in decreased cell proliferation, increased apoptosis, and an increased sensitivity to cisplatin^25^. Further studies have demonstrated that the effectiveness of ULK1 inhibition is enhanced with a combination of mTOR inhibition^23^.

When autophagy was inhibited by targeting VPS34 in our cells, viability and growth were also reduced. Tumor cell death was further enhanced in the presence of BRAFi with the exception of AM38-resistant cells. Inhibition of VPS34 has been previously studied in other cancers. Studies in HER2-amplified breast cancer cells suggested that blocking induced autophagy by VPS34 inhibition may improve antitumor activities of HER2–PI3K inhibitors^40^. In renal tumor cells, VPS34 inhibition by SAR405, a highly potent and selective kinase inhibitor, resulted in tumor cell death under starvation or combined with mTOR inhibition^41^.

As with studies showing synergy between ULK1 and VPS34 inhibitors with mTOR inhibition, our data would support the increased efficacy of autophagy inhibition via ULK1 and VPS34 in CNS tumor cells under stressed conditions. It is interesting to note that under nutrient-poor conditions, BRAFi and autophagy inhibition greatly reduced tumor cell death in all cell lines, including AM38-resistant cells which did not show this effect under standard growth conditions. This is important clinically as solid tumors, including brain tumors, are often under stress including nutrient deprivation and hypoxic conditions^3^. Previous studies using HCQ were limited by the inability of this drug to effectively block autophagy under acidic conditions, such as the central area of a solid mass^31^. Inhibitors that synergize with mTOR inhibition, which would be predicted to be present in starved, hypoxic solid tumors, could increase the benefit of these medications^23,41^.

Overall, our data suggest that early-stage autophagy inhibition is a potential therapy option for autophagy-dependent CNS tumors. These data also demonstrate how environmental factors such as nutrient stress can affect tumor cell response to these treatments. In combination with our previously reported CQ data^18^, this would support the concept that it is the process of autophagy that is important in BRAF^V600E^ CNS tumor cells, not where we target the process. This is important as CQ and HCQ are currently available, FDA approved, and have a long history of patient use with known toxicities. Further pharmacologic optimization of early-stage autophagy inhibitors is ongoing. While the future use of early-stage inhibitors could be promising in the future treatments of BRAF^V600E^ tumors, clinical trials by using CQ or HCQ are currently ongoing. Further preclinical evaluation directly comparing early- and late-stage inhibition will help to define the best method of autophagy inhibition to pursue clinically in autophagy-dependent BRAF^V600E^ CNS tumors.

## Supplementary information

Supplemental FiguresM

Supplementary figure legends
