# Supplementary material for: Effect of early-stage autophagy inhibition in BRAFV600E autophagy-dependent brain tumor cells
Source: Cell Death Dis. 2019 Sep 12;10(9):679. doi: 10.1038/s41419-019-1880-y (PMC6742667; doi:10.1038/s41419-019-1880-y)

Supplementary Figure 1

(A)

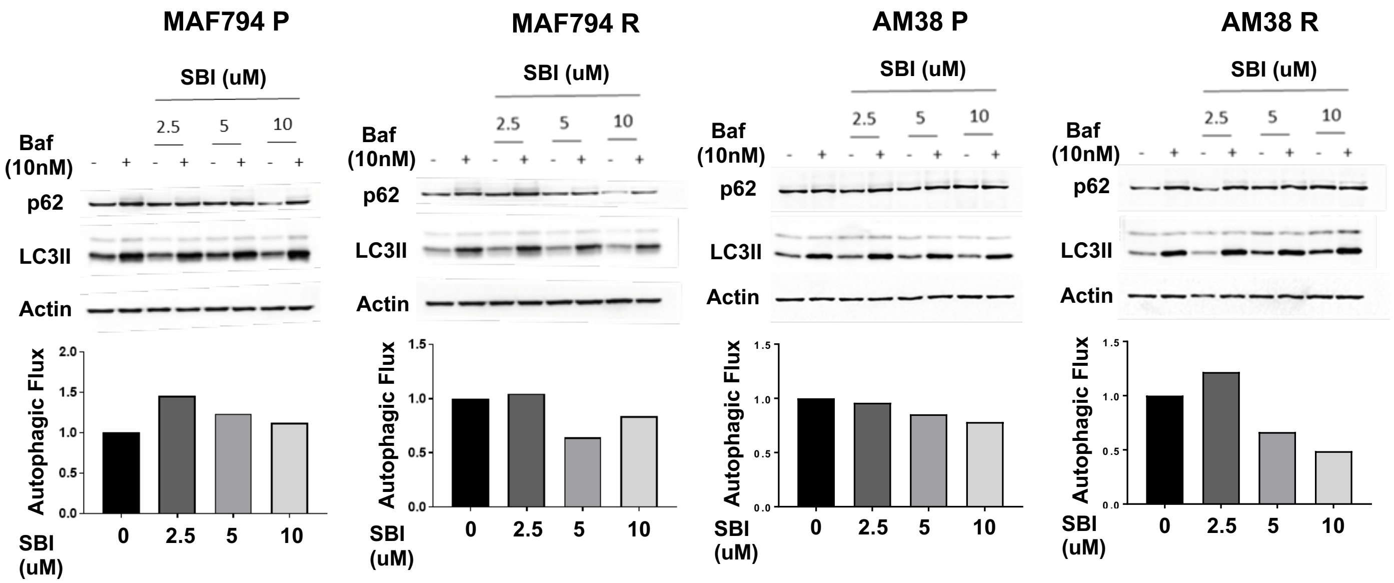

(B)

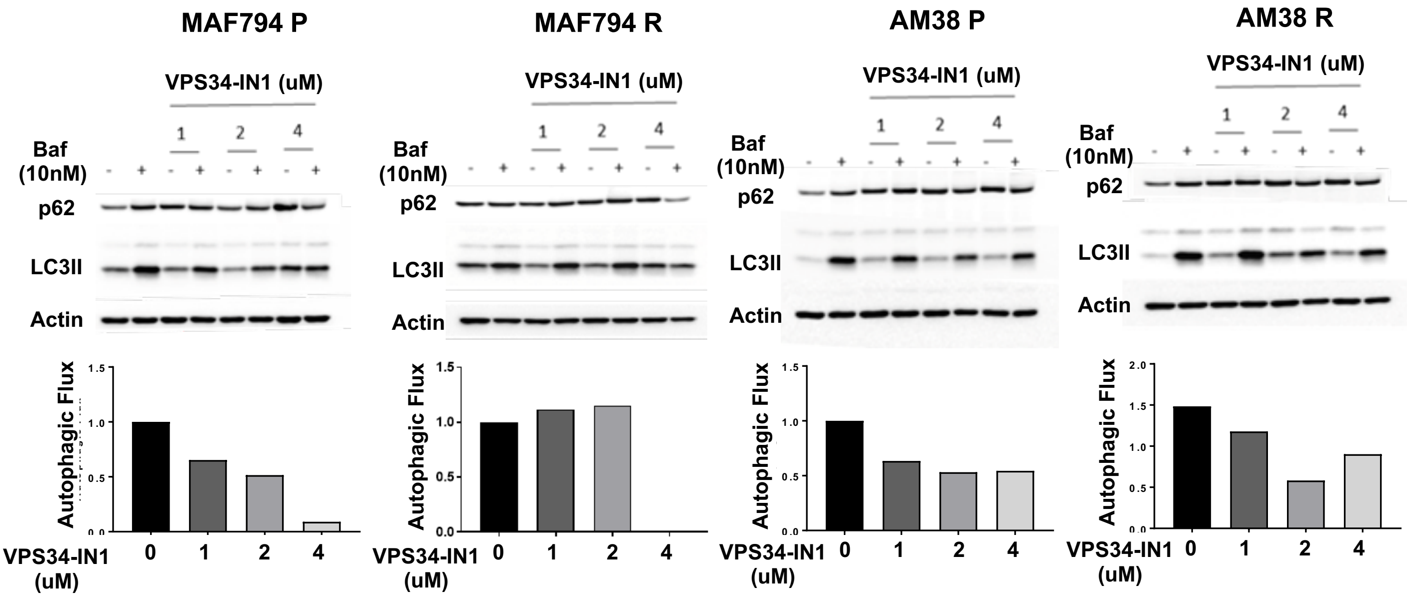

Supplementary Figure 2

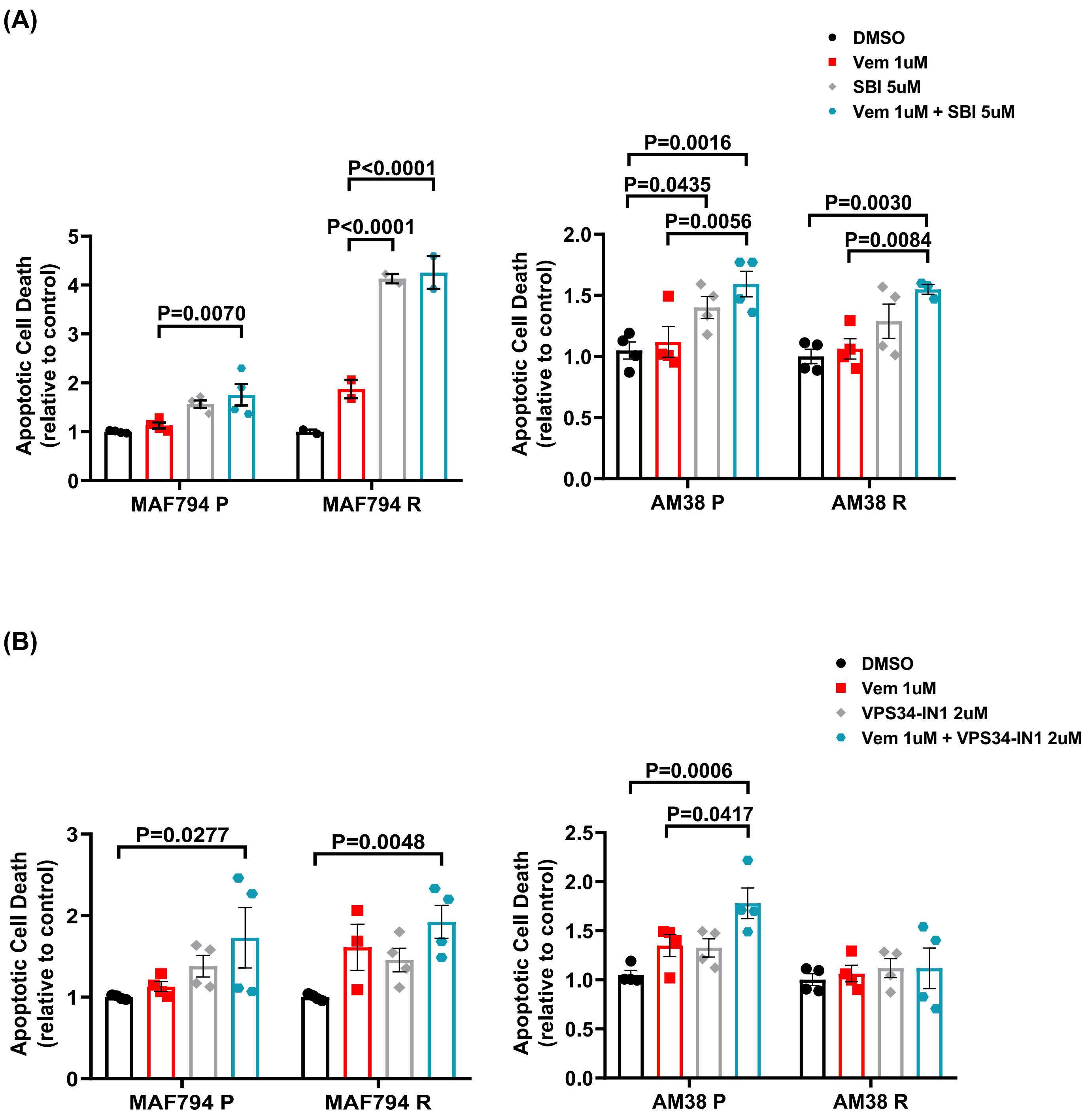

Supplementary Figure 3

(A)

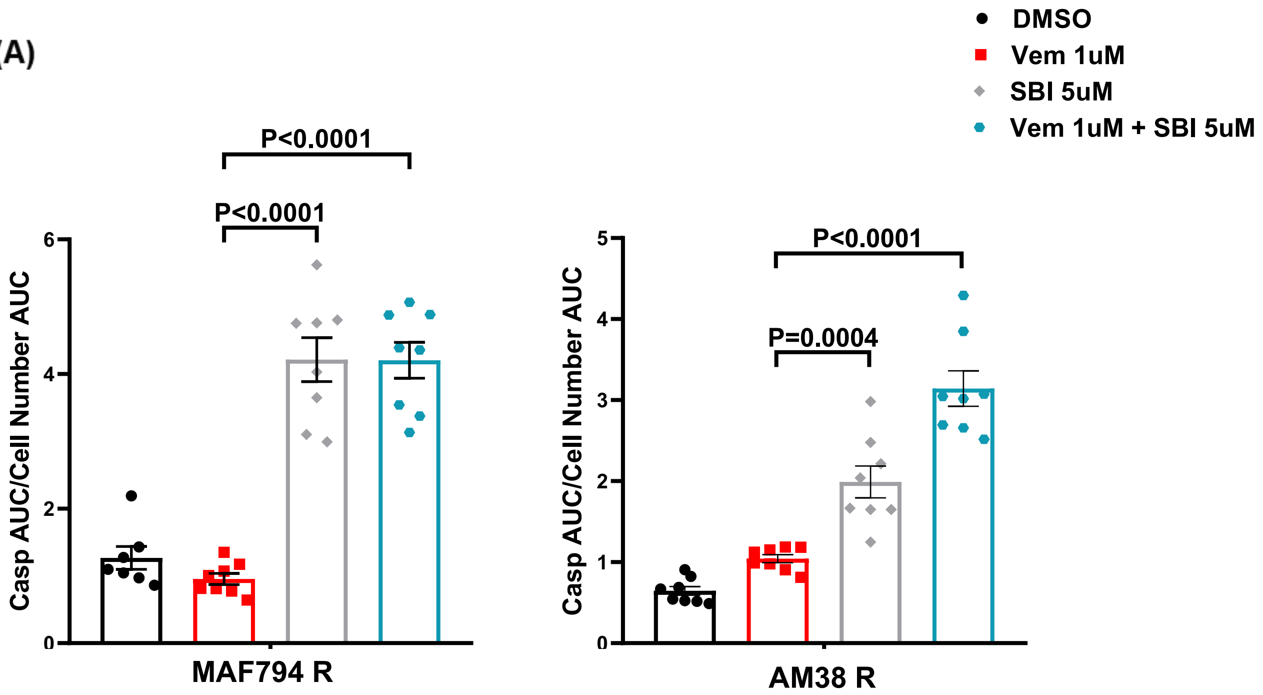

(B)

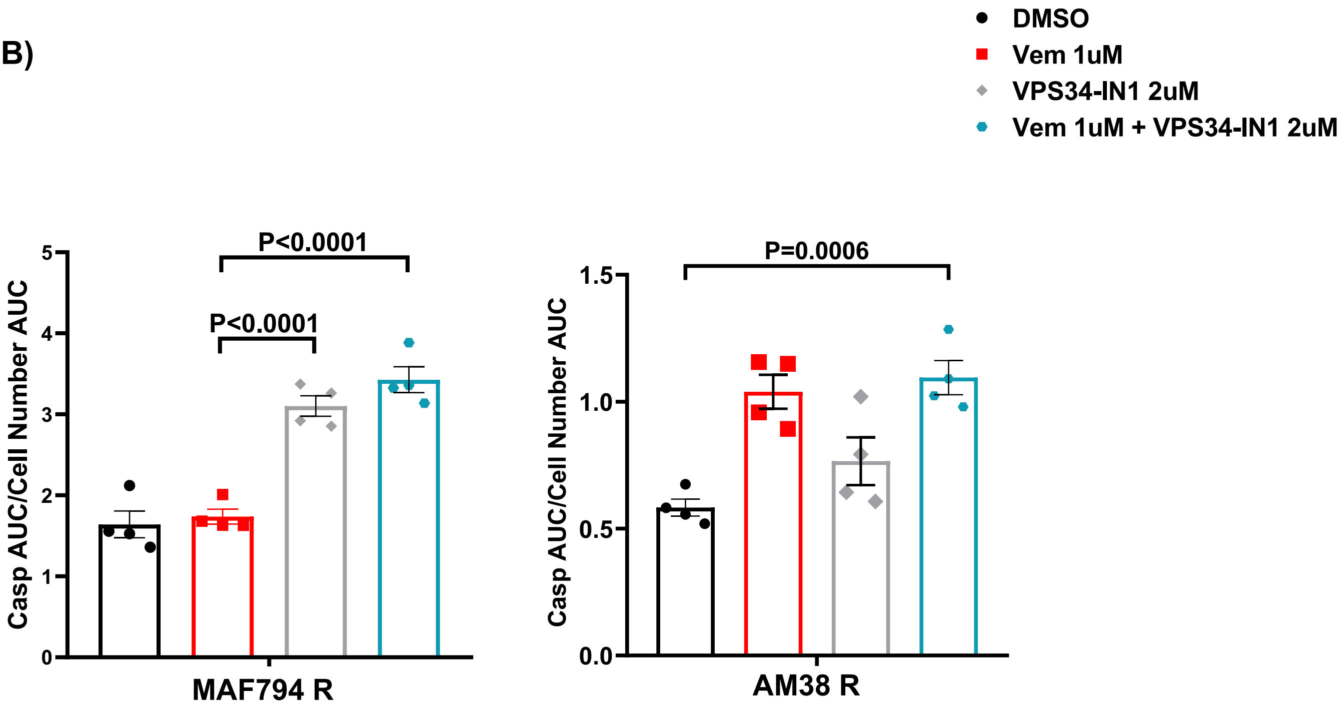

Supplementary Figure 4

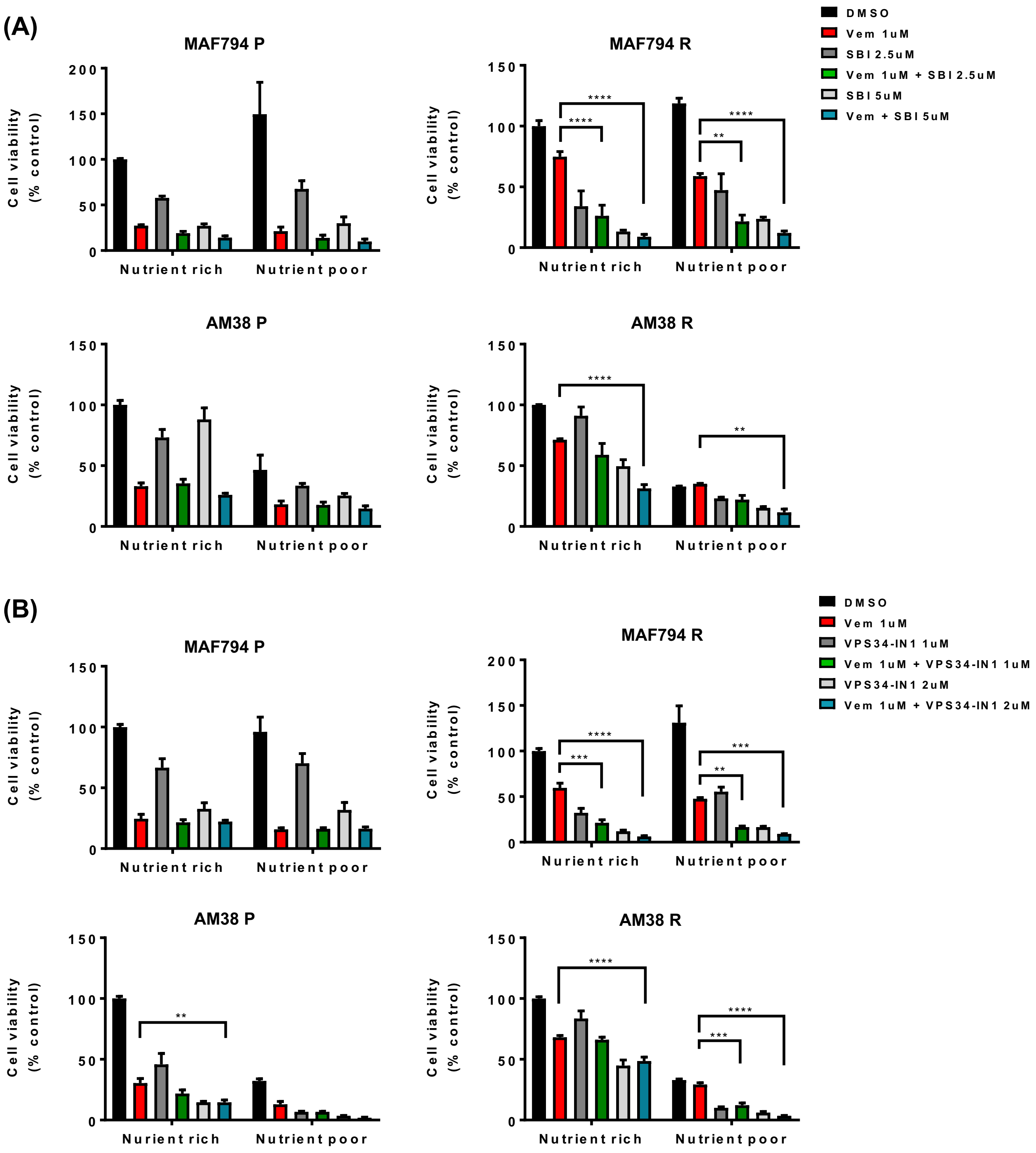

Supplementary Figure 5

(A)

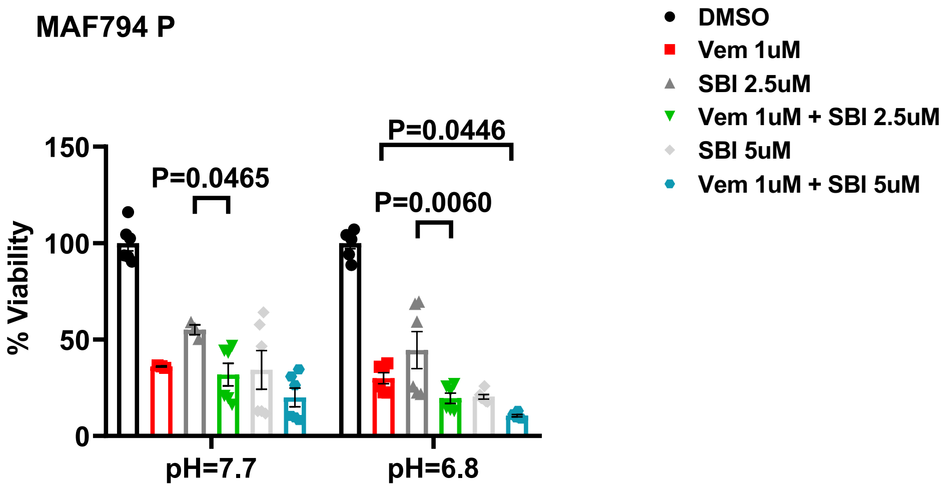

(B)

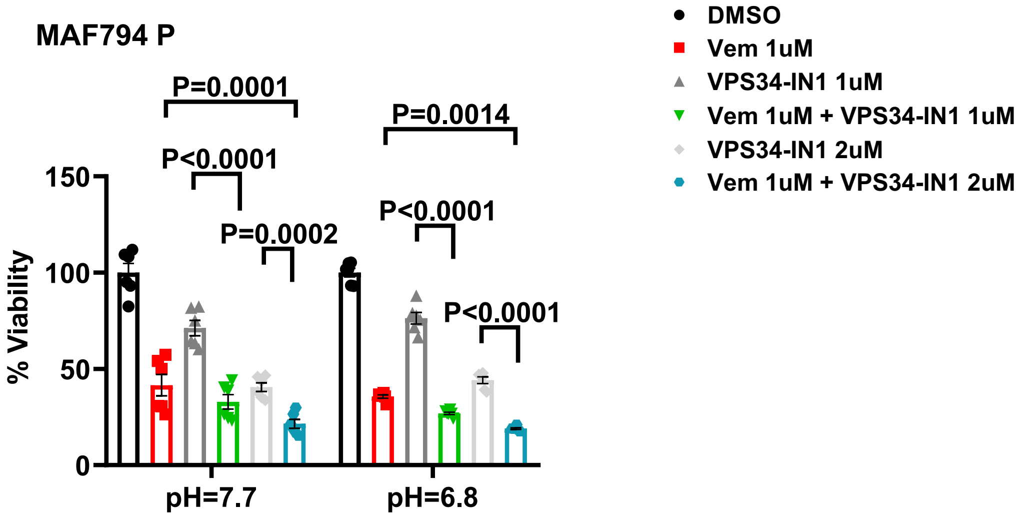

(C)

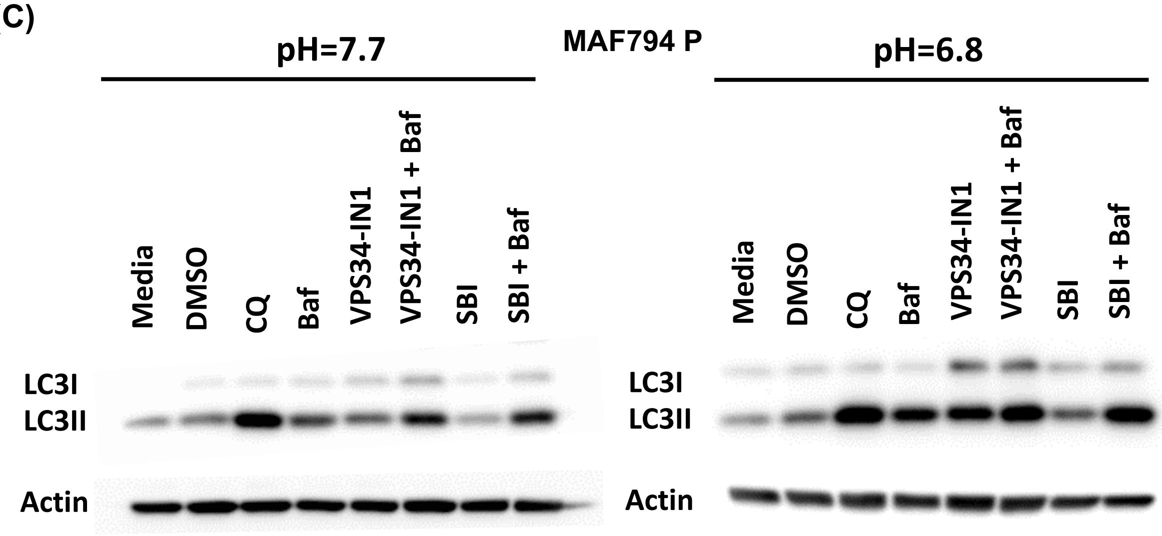

Supplementary Figure 6

(A)

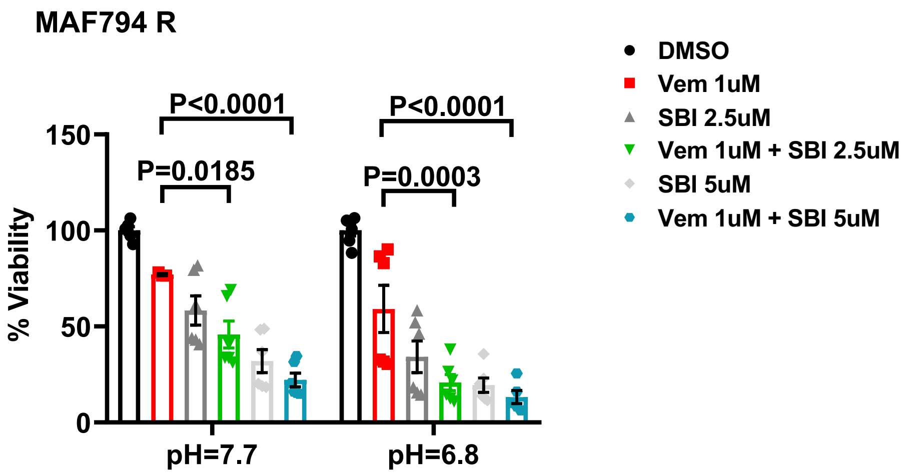

(B)

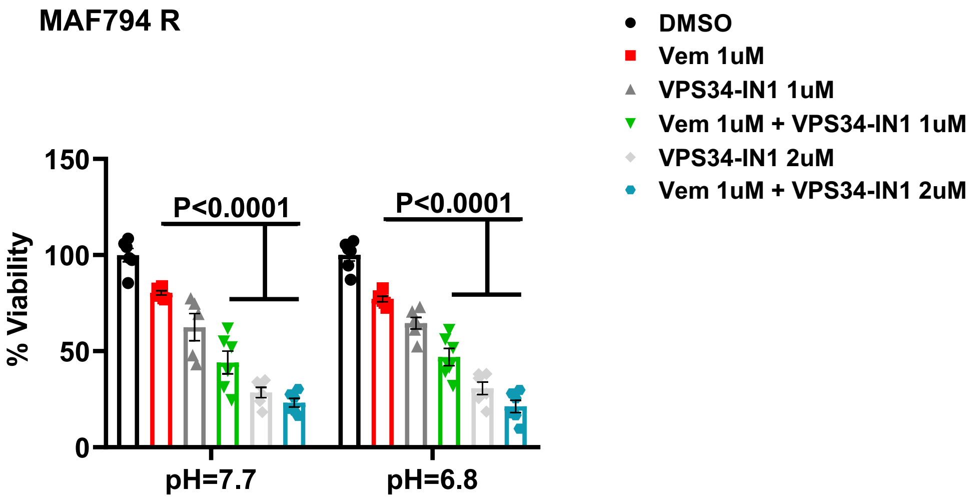

(C)

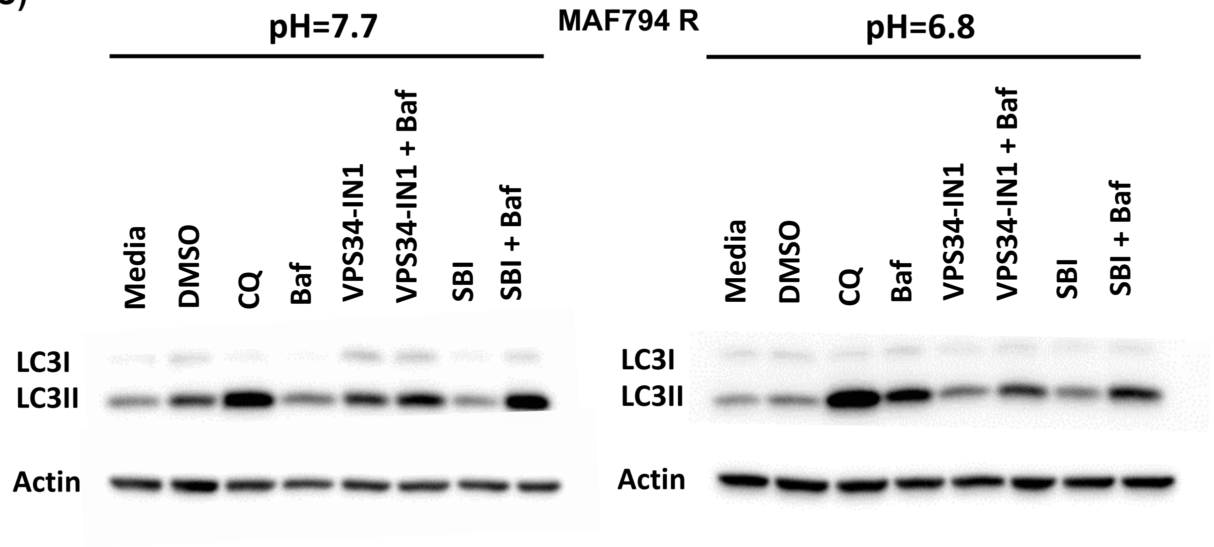

Supplementary Figure 7

(A)

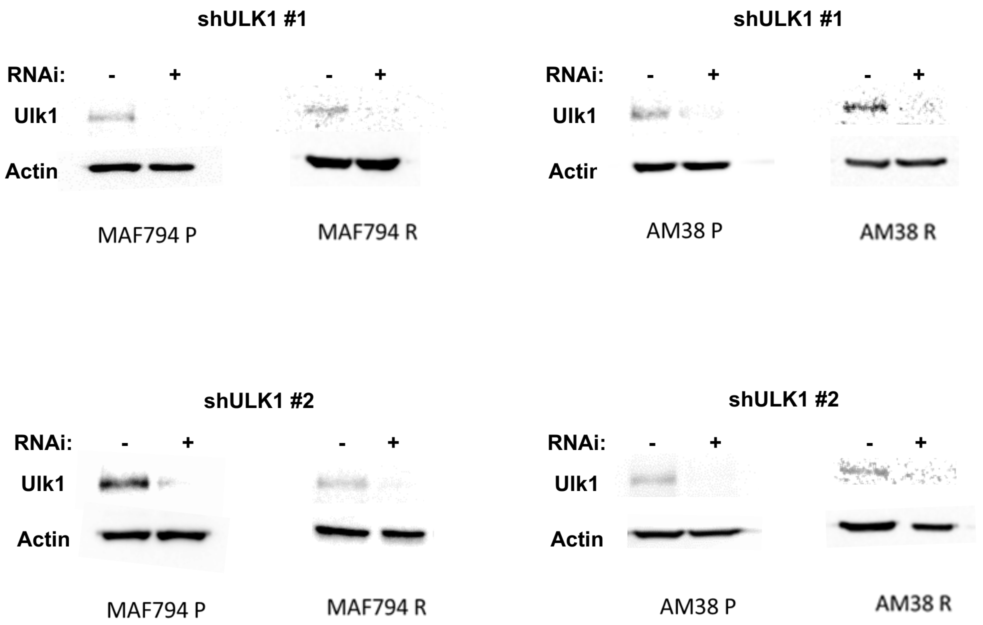

(B)

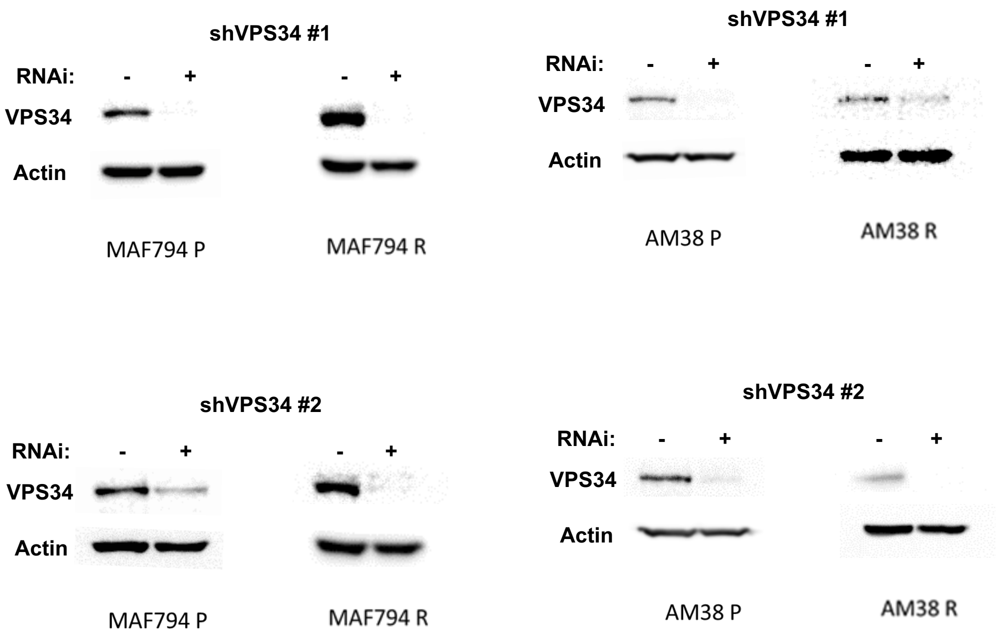

Supplementary Figure 8

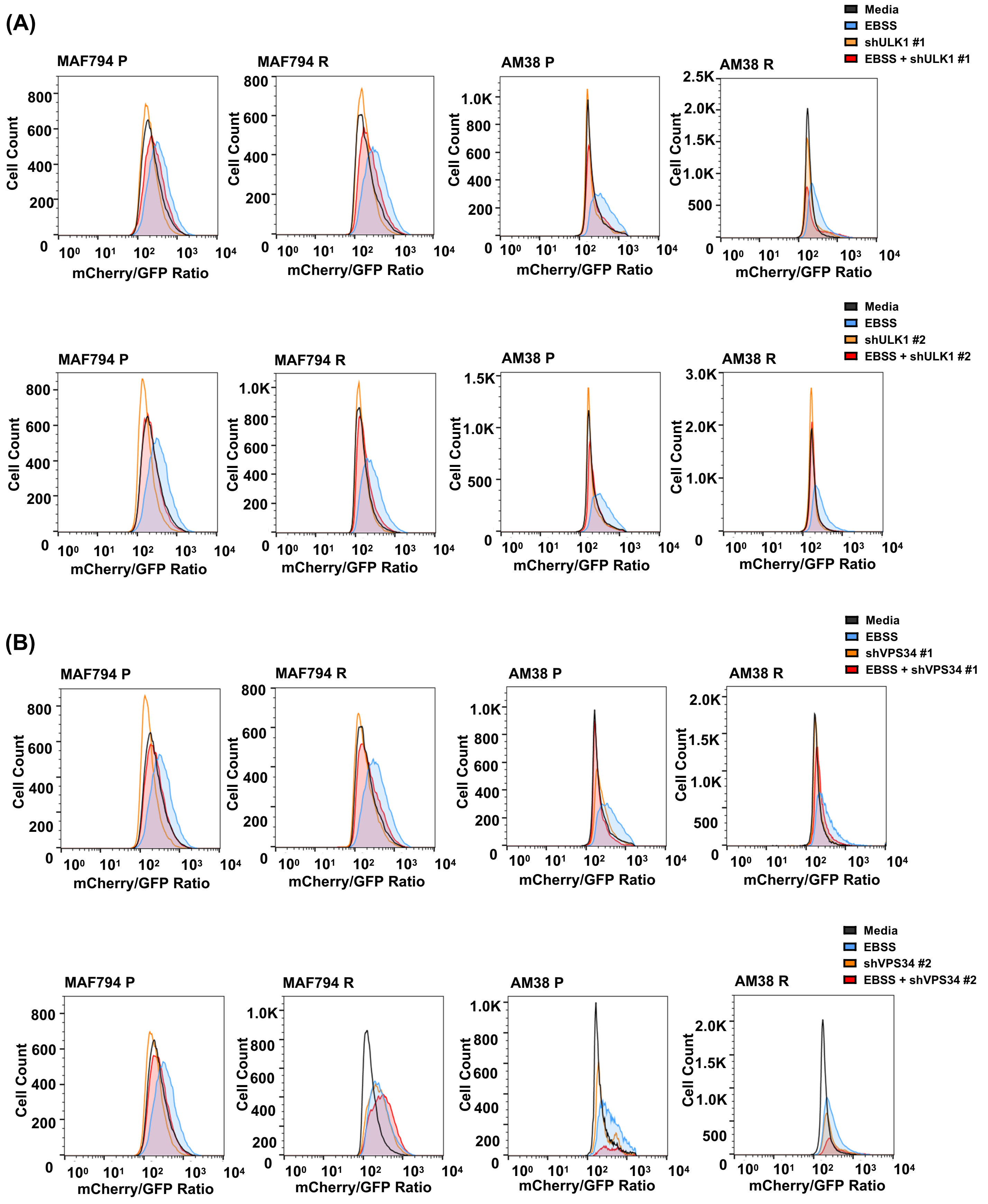

Supplementary Figure 9

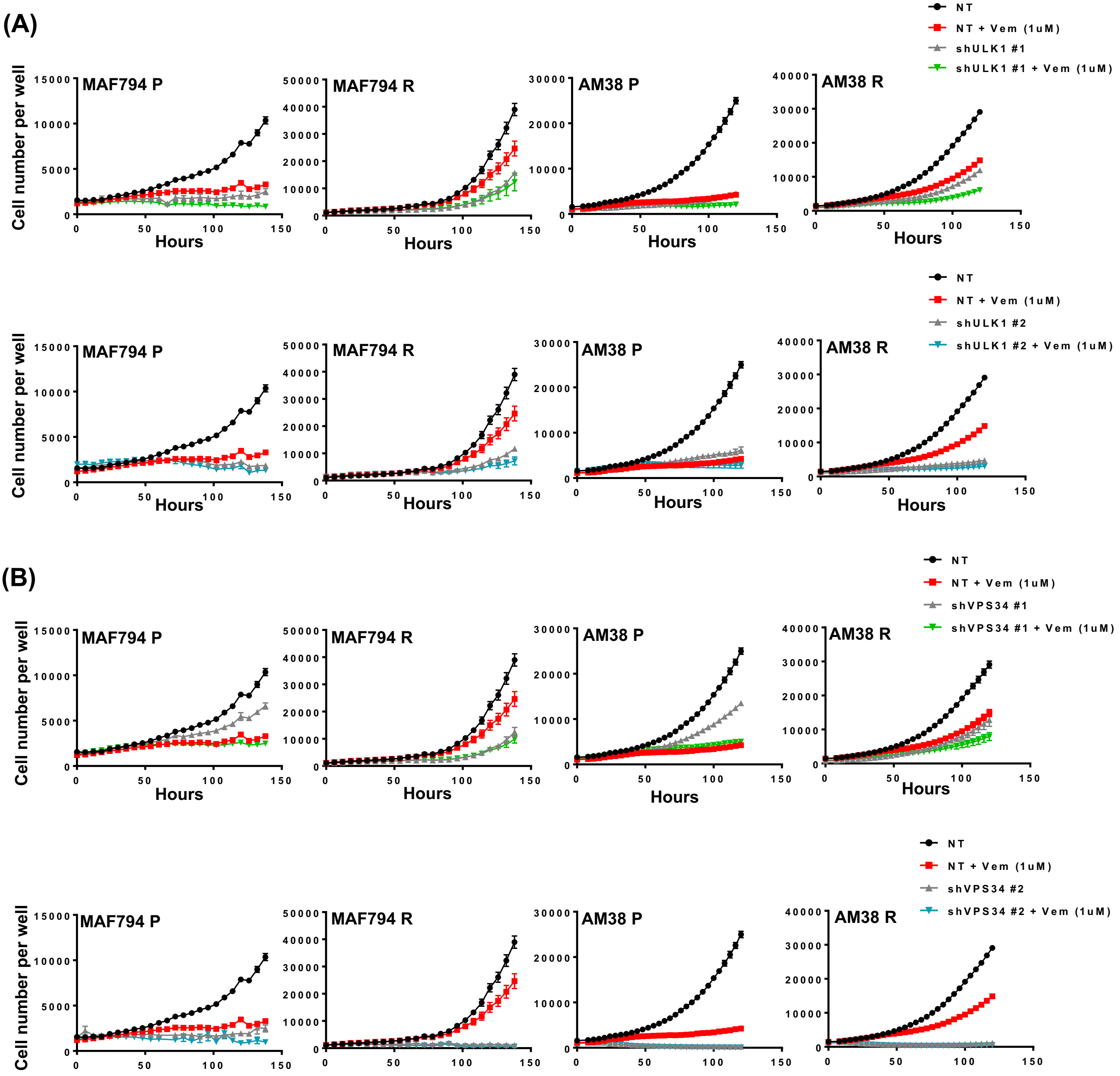

Supplement: Supplementary file 1 — Supplemental FiguresM [file 41419_2019_1880_MOESM1_ESM.pdf]
