# Supplementary material for: Effect of early-stage autophagy inhibition in BRAFV600E autophagy-dependent brain tumor cells
Source: Cell Death Dis. 2019 Sep 12;10(9):679. doi: 10.1038/s41419-019-1880-y (PMC6742667; doi:10.1038/s41419-019-1880-y)
Supplement: Supplementary file 2 — Supplementary figure legends [file 41419_2019_1880_MOESM2_ESM.docx]

**Supplementary Figure Legends**

**Supplementary Figure 1. SBI or VPS34-IN1 decreases autophagy in parental and resistant MAF794 and AM38 cells.** Representative westerns and quantification of samples demonstrating reduced autophagic flux following SBI **(A)** or VPS34-IN1 **(B)** treatments for 6h and 24h respectively with or without bafilomycin (10nM) (n=2).

**Supplementary Figure 2. Pharmacologic early stage autophagy inhibition causes apoptotic cell death in parental and resistant cells.** Percent apoptotic cell death as measured by annexin positive cells was obtained following a 48h treatment of cells with SBI **(A)** or VPS34-IN1 **(B)** in the presence or absence of BRAFi using Guava EasyCyte flow cytometer (Luminex Corporation). Combined early (Annexin V+/7-AAD -) and late (Annexin V+/7-AAD+) apoptotic cell death is represented as a relative value compared to control. Dunnett’s multiple comparisons; mean ± s.e.m (n=2). *p<0.05.

**Supplementary Figure 3. Early stage autophagy inhibition results in caspase-dependent apoptosis in resistant cells.** Cells were treated with SBI **(A)** or VPS34-IN1 **(B)** in the presence or absence of BRAFi followed by the addition of CellEvent caspase-3/7 Green reagent. Cells were monitored using IncucyteZOOM. The area under the curve (AUC) of caspase activation over a 5 day treatment period, measured by the number of green objects per well, was normalized to the AUC of cells per well. Dunnett’s multiple comparisons; mean ± s.e.m (n=2). *p<0.05.

**Supplementary Figure 4. Nutrient poor conditions result in improved response to early stage autophagy inhibition.** Percent cell viability following the treatment of BRAF^V600E^ parental and resistant cells with SBI **(A)** or VPS34-IN1 **(B)** in the presence or absence of BRAFi under nutrient rich (standard media) and poor (75% reduced-serum media) conditions. Dunnett’s multiple comparisons; mean ± s.e.m (n=2). *p<0.05.

**Supplementary Figure 5. Acidic conditions do not affect the activity of autophagy inhibitors in MAF794P BRAF^V600E^ brain tumor cells.** Percent cell viability as measured by CellTiter Glo (compared to control DMSO) following a 5-day exposure to SBI **(A)** or VPS34-IN1 **(B)** in the presence or absence of BRAFi under normal (pH=7.7) and acidic (pH=6.8) conditions. Dunnett’s multiple comparisons; mean ± s.e.m (n=2). *p<0.05. **(C)** Representative western blots demonstrating autophagic flux under both normal and acidic conditions following exposure to autophagy inhibitors as indicated.

**Supplementary Figure 6. Acidic conditions do not affect the activity of autophagy inhibitors in MAF794R BRAF^V600E^ brain tumor cells.** Percent cell viability as measured by CellTiter Glo (compared to control DMSO) following a 5-day exposure to SBI **(A)** or VPS34-IN1 **(B)** in the presence or absence of BRAFi under normal (pH=7.7) and acidic (pH=6.8) conditions. Dunnett’s multiple comparisons; mean ± s.e.m (n=2). *p<0.05. **(C)** Representative western blots demonstrating autophagic flux under both normal and acidic conditions following exposure to autophagy inhibitors as indicated.

**Supplementary Figure 7. Verification of ULK1 and VPS34 knockdowns in BRAF^V600E^ parental and resistant cells.** Representative westerns showing the effectiveness of ULK1 **(A)** and VPS34 **(B)** RNAi in parental and resistant MAF794 and AM38 cells.

**Supplementary Figure 8. Genetic inhibition of ULK1 and VPS34 reduces induced autophagic flux in BRAF^V600E^ cells.** Representative histograms demonstrating basal and induced autophagic flux in the presence or absence of ULK1 **(A)** and VPS34 **(B)** RNAi in parental and resistant MAF794 and AM38 cells.

**Supplementary Figure 9. Early stage genetic inhibition of autophagy diminishes growth and improves effectiveness of BRAFi response in resistant cells.** Growth curves of MAF794 and AM38 parental and resistant cells following the genetic inhibition of ULK1 **(A)** and VPS34 **(B)** in the presence or absence of BRAFi. Cell number per well was obtained overtime using Incucyte ZOOM (Essence Bioscience). (mean ±s.e.m., n=2)
